# Early Diagnosis of Tubercular Meningitis in Children

**Published:** 1888-03

**Authors:** H. L. Elsner

**Affiliations:** Professor of Clinical Medicine, Syracuse Medical College


THE BUFFALO MEDICAL AND SURGICAL JOURNAL

Vol. XXVII. MARCH, 1888. No. 8

### Original Communications.

### *EARLY DIAGNOSIS OF TUBERCULAR MENINGITIS IN CHILDREN.*

By H. L. ELSNER,

Professor of Clinical Medicine, Syracuse Medical College.

The early diagnosis and prognosing of any given case is a source of great satisfaction and profit to the physician as well as to the patient. This must necessarily be so for a large number of reasons, which flash through our minds almost simultaneously. If the physician can, during the prodromal stage of a disease, accurately diagnose and prognose, he not only benefits the patient, but aids in raising the standard of his profession. There are many diseases which begin with similar symptoms, yet the subsequent course of each is different. It is often not only difficult, but impossible, to diagnose a case accurately during the first days of attendance. By carefully grouping symptoms, by a process of elimination and a knowledge of the previous history of our cases, we are better able to diagnose correctly. It will be my object in this paper to give you a few hints and symptoms which will aid in making an early diagnosis of cerebral tuberculosis. When we consider the comparatively large number of cases that we are called upon to treat, the fatality of the malady, and the chronic course of the disease in many instances, we will at once agree that any symptoms which aid in early diagnosis require our careful attention and closest study. In giving the symptoms which have seemed to me sufficient for an early recognition of the disease, I am not drawing from the experience of others, but am giving the conclusions which have been deduced from a careful consideration of the cases seen in private practice. It matters little whether or not we accept, as proven, that a deposit of tubercles at the base of the brain cannot give rise to symptoms before inflammatory action takes place in the piamater, or that simple tubercular deposit is sufficient to cause symptoms without meningitis. The fact remains, that in my cases there have been symptoms of disease before the meningitis proper manifested itself by an uninterrupted train of symptoms. The presence of any one symptom *alone* would not justify us in making the diagnosis of tubercular meningitis. It is in this disease, as in all others, a consideration of all that the case presents, that justifies a diagnosis. These symptoms are to be considered as prodromata, not as symptoms of the disease after it has manifested itself openly, and is marching rapidly from the stage of irritation to that of pressure; but as they often appear when they are followed by days of apparent health, only to renew themselves and finally merge into the continuous meningitis, which runs a rapid course and terminates fatally in from fifteen to twenty days. The symptoms to which I wish to call your attention are:

First—Altered disposition.

Second—Headache.

Third—Vomiting and constipation.

Fourth—Cerebral macule.

Fifth—Ptosis and facial paralyses.

Sixth—Convulsions.

Seventh—Pulse and fever.

1. *Altered Disposition.*—For several weeks before the outbreak of the disease, by well-defined symptoms, the mother or nurse, playmate or companion, notice a decided change in the disposition of the child. Whenever a child, who has previously found pleasure in its toys, suddenly, for a day or longer, turns from them, or has moments when they are truly enjoyed, quickly followed by a dislike, manifested by its actions, we may safely (*when coupled with other symptoms*) look for some abnormality. In some cases, older children suddenly, while in the midst of their play, discontinue, lie down for a few moments, and are then again ready to resume. Other children, who have naturally a mild and quiet disposition, become unruly, unmanageable—seem entirely changed. Children who have been affectionate, fond of the caresses of parents, become at times petulant and irritable. In the case of a child nine years of age, who finally died of tubercular meningitis, one of the first early symptoms, and one which greatly annoyed the parents, was a dislike which the patient took to a younger sister with whom she had always played and for whom she had always evinced the strongest affection. In another case, it was useless for the mother to attempt to quiet a previously affectionate and loving boy of twelve, who finally yielded to tubercular meningitis. The only person whom he would mind was a neighbor for whom he had never shown the slightest regard, but who was now given the preference. Altered disposition, taken alone, may not be of much value as a prodromal symptom, but when, added to it, there is marked emaciation and one or more of the symptoms mentioned, it becomes one of great value.

2. *Headache.*—The headache which was found in those children who were old enough to describe their symptoms, was not the same in character in all cases. I have frequently watched children, to note the expression of their face during the hydrocephalic cry, and have come to the conclusion that it is caused by a pain of a shooting or darting character. If you will watch the facies of a child during the cry, I am satisfied that you will agree with me in my conclusion. Frequently, older children have for weeks before the manifestation of continuous meningeal symptoms complained of a very severe and acute cephalalgia. Often you will find the only early symptom in a case the severe headache, which may last for several hours, followed by days or weeks of health, to be renewed at shorter intervals. Some describe a darting pain, quickly put their hand to the head, and as quickly appear perfectly normal, and in a few moments are again deeply interested with their playmates. A number of cases will offer as their only early symptoms acute and passing cephalalgia, followed by characteristic vomiting, which I have learned to consider almost pathognomonic of brain trouble, and which will be explained in the following pages. I think that I am safe in saying that persistent or recurring headaches in children, with normal temperature, when not dependent upon errors in diet or other explainable transient causes, and when accompanied with vomiting or other symptoms enumerated above, partaking of the character of the headaches described, are to be looked upon with suspicion, and meningeal tuberculosis may be expected in a large proportion of the cases.

3. *Vomiting and Constipation.*—Among the most frequent early symptoms of tubercular meningitis, we find vomiting and constipation. It is to the first of these symptoms that I wish more particularly to call your attention. The vomiting which we find as an early symptom is characteristic; it is one of the symptoms of greatest import in making the diagnosis during the prodromal period. I think that my readers will agree with me, after watching their cases to note this symptom and the manner in which it manifests itself, that from it alone can the early diagnosis be made in eighty per cent. of all cases in children. Now, let me tell you what we consider almost characteristic of this vomiting. A child, previously healthy, without any error in diet, *suddenly*, and without warning, vomits large quantities of fluid and mucus without color, but watery. No sooner has the child vomited, than it is ready to run along to its play; or if it suddenly awakens to vomit, it readily falls back into a natural sleep. The parents are usually surprised to find the child vomit, for they can attribute it to no error of diet or any assignable cause. The child will probably, after the first emesis, continue enjoying (to all appearances) perfect health, until in the course of from three to five or seven days it again, without one moment's nausea or any expression of sickness, vomits the same large quantities, and seems entirely unaffected by the act, but ready to resume its occupation. If you have noticed children before they eject the contents of their stomachs when suffering from indigestion, you will find at first a pallor, the body covered with perspiration; in fact, you find all the attendants of nausea preceding the act of vomiting. Vomiting is followed by a relaxed and enfeebled state, during which the child wishes to rest, and its color is changed. In the vomiting of brain lesions in children, a different state of affairs is found. The vomiting is not preceded (as a rule) by symptoms of nausea. The child, while playing or during its sleep, while we are looking into its face and are not able to notice the slightest change in expression, without retching, suddenly throws up large quantities of fluid, and at first seems entirely unaffected. During the prodromal stage, you will frequently be consulted by parents, and asked to relieve this vomiting. The history of a large number of cases could be given where I have been asked to relieve this symptom, and in which the diagnosis of tubercular meningitis in its incipiency was made, giving a grave prognosis. It has become routine practice with me, after getting the previous history, to ask every parent presenting a child for treatment of vomiting as the foremost symptom, whether or not the child is sick long before vomiting, whether the child vomits unexpectedly, and what change is noticed in the condition of the child after vomiting. Given the case of a child who, without previous sickness, nausea or error in diet, vomits *suddenly, possibly repeatedly*, each time *without precediug sickness*, followed by a return to the same condition in which the child was found before the act, with or without some of the symptoms above mentioned, we may strongly suspect incipient meningeal tuberculosis. If this characteristic symptom is accompanied by constipation, we have an additional symptom which aids us in diagnosis. *Constipation*, as an early symptom, is only of value when associated with other symptoms. Children are prone at times to suffer from constipation, and it is not a symptom that will justify us in making a diagnosis. If, however, a child presents who is constipated, at the same time has "meningeal vomiting" (allow me to suggest the term), and possibly one or more of the symptoms above mentioned, there could be no hesitation in making a diagnosis of meningeal tuberculosis in its incipiency.

4. *Cerebral Macule.*—At the Poliklinik, in Vienna, it was routine practice with Monti to run his finger-nail along the skin of a child in whom he suspected brain trouble, and await the resulting macule. If the redness resulting was no more than it would be in a healthy child, and faded rapidly, he would exclude tubercular meningitis, but if the skin was suffused with a bright, red tint, and this remained for from five to ten or even fifteen minutes, he would turn to the class and invariably say, "*prognosis infausta est.*" The cerebral macule is a symptom which Trosseau brought most prominently before the profession, and one which he considered of the greatest importance, yet not always pathognomonic of "cerebral fever." I cannot describe it better than to use the words of this renowned teacher: "When I gently made on her abdomen (speaking of a case), with my nail, cross markings, longitudinally and transversely, in less than half a minute the portion of skin which I had touched was suffused with a very bright, red tint, which was diffused at first, but grew by degrees fainter, leaving lines along the track where the nail had passed, lines of a deeper red color, which persisted for a pretty long time." "This is what I mean by cerebral macule." This cerebral macule is not always to be considered as a certain and unfailing symptom of tubercular meningitis, but I am satisfied that it is present in the larger number of cases. In many cases, it has materially aided me in making an early diagnosis. In August, 1879, was called to see a male child, two years old, who, without warning, was suddenly seized with convulsions, severe, and involving all extremities alike. In spite of the usual remedies, including ether, these convulsions continued for three hours. At this time, the cerebral macule was well marked; so well, indeed, that it was plainly noticeable wherever the child was touched. For three months after these convulsions, the child was, to all appearances, perfectly healthy. At the end of this time, it was brought to me with the characteristic meningeal vomiting, cerebral macule well marked; gave a grave prognosis and diagnosed incipient tubercular meningitis. For six months after this time I observed the child, the cerebral macule was always present; finally, the child passed into the hands of another physician, but it died about twelve months after the first convulsion, of tubercular meningitis. In looking over my notes, I find many cases in which I was enabled, at my first visit, to make a diagnosis and prognosis of these cases, feeling justified in doing so after considering the symptoms, including the cerebral macule. You naturally ask: "Does this symptom show itself in any other disease?" It is occasionally noticed in other diseases: among them, broncho-pneumonia, with marked meningeal symptoms; also in cases where the diagnosis of meningeal congestion had been made. It is only exceptionally present in other than cerebral fevers. I have always looked for this symptom, and have, as a rule, found it present in the early days of tubercular meningitis. In the differentiation of typhoid fever with brain symptoms from tubercular meningitis, the presence of this symptom is invaluable in making the diagnosis.

5. *Ptosis and other Paralyses* (Facial).—In a few cases which have come under my observation, ptosis was the first symptom which was noticeable. I well remember the case in which I first found this symptom. In the winter of 1878, I was called to see a lady who was in the last stages of tubercular consumption, treated her for some weeks, when, one day, the baby, a female, sixteen months old, was brought into the room for examination. The father said that the child was well, but could not keep its left eye open. He thought the "upper lid too long." Upon examining the child carefully, I was unable to find other symptoms that would aid in making a diagnosis. In a few weeks, there was ptosis of the right lid also. Then followed a time during which the child could only see when its lids were raised by others. In six months from the time of the first ptosis, the child died of tubercular meningitis. In the case of a girl, ten years of age, ptosis was associated with headache, vomiting, and the cerebral macule. She died on the seventeenth day of the disease. Paralyses in children in the domain of the motor oculi, facial and hypoglossal, arising gradually with or without other symptoms of brain disease, are of the greatest importance, and are usually followed by tubercular meningitis. Though I have watched the condition of the pupils, I don't believe that they present any features which assist in the diagnosis of the disease under consideration.

6. *Convulsions.*—The fact that a child has convulsions, when taken alone, is of no value in making the diagnosis. Of all the children that we are called upon to attend during convulsions, only a small proportion succumb to tubercular meningitis. In a few cases, we do find convulsions during the prodromal period; they are exceedingly rebellious and likely to recur. In these cases, there is no error in diet or other causes than tubercular deposit. In the case above mentioned, there must have been a tubercular deposit, giving rise to the convulsions twelve months before the child died, and almost as many months before the appearance of continuous symptoms. From anatomical appearances of the brains of children who have died before the manifestation of symptoms referable to meningitis, but after convulsions, the tubercular deposit has been found in some at the base, in others upon the convexity of the brain. In the majority of the cases which the writer has studied, convulsions were not present, as a rule, during the prodromal period; and when they were, they were associated with other symptoms which aided in making the diagnosis easy.

7. *Pulse and Fever.*—With regard to pulse and temperature, I do not believe that I can give anything characteristic. In some cases, the pulse is slow, in others irregular, and in another class of cases very rapid. As a rule, the temperature does not run high during the prodromal stage, and when these symptoms which we have considered appear many weeks before the final and continuous illness, the temperature during that time is not actually above 100° Fahrenheit. There are cases in which, for several days, we find elevation of temperature with all other symptoms of a rapidly fatal tubercular meningitis; suddenly, a decided fall, disappearance of the symptoms, and a return to apparent health. We begin to doubt the correctness of our diagnosis, when suddenly there is a fresh outbreak of all the symptoms, increased in severity, leading to a fatal termination. The end is caused by paralysis of the centers in the medulla oblongata. The appearance of tubercular meningitis *without* prodromal symptoms appears to me to be exceptional. That we do have these cases, I cannot deny, but they are *exceedingly rare*. There are other symptoms than those which have been mentioned that aid us in making up the picture of the disease, but to these I wished to call your attention and invite a careful study of their relative importance. It would be unfortunate, indeed, for us to treat a case of incipient cerebral tuberculosis for simple gastric indigestion; yet this mistake is frequently made. There is not one pathognomonic symptom of the disease under consideration, but we may conclude that the association of several of the symptoms point strongly to the true seat and character of the disease. The eloquent words of Trousseau again occur to me:

"We must not look at a portion of the picture only, but at the whole at once. In order to know the drama well, the whole play must be seen, and not one scene alone."

